# Supplementary material for: The efficacy of current treatment processes to remove, inactivate, or reduce environmental bloom-forming Escherichia coli
Source: Microbiol Spectr. 2024 Jul 9;12(8):e00856-24. doi: 10.1128/spectrum.00856-24 (PMC11302305; doi:10.1128/spectrum.00856-24)
Supplement: Supplemental material — Tables S1 and S2. [file spectrum.00856-24-s0001.pdf]

Supplementary Information

Supplementary Table 1: Happy Valley Reservoir water quality chemistry parameters before (raw) and after (settled) jar test and filtration for six 'starved' strains tested.

	**B1_1330_2_1_ WA_Bloom**	**B1_1863_3_1_ WA_Bloom**	**A_E258_A1_ Bloom_K16**	**C_H218_non-bloom_KL16**	**A_H386_non-bloom**	**B1_B226_non-bloom**
Turbidity (NTU) - Raw	12.80 ± 0.35	12.80 ± 0.35	12.80 ± 0.35	12.80 ± 0.35	12.80 ± 0.35	12.80 ± 0.35
Turbidity (NTU) - Settled	2.33 ± 1.15	2.40 ± 0.56	2.40 ± 1.40	2.23 ± 1.06	2.80 ± 1.50	2.47 ± 1.14
% turbidity removal	82 ± 9	81 ± 4	81 ± 11	83 ± 8	78 ± 11	81 ± 9
Colour (HU) 400nm - Raw	47 ± 3	47 ± 3	47 ± 3	47 ± 3	47 ± 3	47 ± 3
Colour (HU) 400nm - Settled	6 ± 0	6 ± 0	6 ± 0	6 ± 0	6 ± 0	6 ± 0
% colour removal	87± 1	87± 1	87± 1	87± 1	87± 1	87± 1
UV abs 254nm (cm^{-1}) -Raw	0.29 ± 0.01	0.29 ± 0.01	0.29 ± 0.01	0.29 ± 0.01	0.29 ± 0.01	0.29 ± 0.01
UV abs 254nm (cm^{-1}) - Settled	0.087 ± 0.002	0.086 ± 0.003	0.087 ± 0.002	0.086 ± 0.002	0.087 ± 0.001	0.087 ± 0.001
% UV abs removal	71± 1	71± 1	71± 1	71± 1	70 ± 1	70 ± 1
DOC (mg/L) - Raw	8.2 ± 0.2	8.2 ± 0.2	8.2 ± 0.2	8.2 ± 0.2	8.2 ± 0.2	8.2 ± 0.2
DOC (mg/L) - Settled	4.50 ± 0.5	4.53 ± 0.67	4.37 ± 0.21	4.20 ± 0.10	4.33 ± 0.25	4.30 ± 0.26
% DOC removal	46 ± 7	45 ± 9	47 ± 3	49 ± 2	47 ± 4	48 ± 5

***Data shows the mean and standards deviation from three replicate experiments.**

Supplementary Table 2: Prospect Reservoir water quality chemistry parameters before (raw) and after (settled) after jar test for six 'starved' strains tested.

	**B1_1330_2_1_ WA_Bloom**	**B1_1863_3_1_ WA_Bloom**	**A_E258_A1_ Bloom_K16**	**C_H218_non-bloom_KL16**	**A_H386_non-bloom**	**B1_B226_non-bloom**
Turbidity (NTU) - Raw	1.93 ± 0.46	1.93 ± 0.46	1.93 ± 0.46	1.93 ± 0.46	1.93 ± 0.46	1.93 ± 0.46
Turbidity (NTU) - Settled	1.60 ± 0.40	1.60 ± 0.17	1.43 ± 0.21	1.50 ± 0.26	1.57 ± 0.25	1.53 ± 0.21
% turbidity removal	10 ± 47	14 ± 20	22 ± 27	20 ± 21	15 ± 28	16 ± 33
Colour (HU) 400nm - Raw	7	7	7	7	7	7
Colour (HU) 400nm - Settled	6 ± 1	5 ± 0.00	5.67 ± 0.58	5.33 ± 0.58	5.67 ± 0.58	5.67 ± 0.58
% colour removal	19 ± 8	28 ± 0	19 ± 8	23 ± 8	19 ± 8	19 ± 8
UV abs 254nm (cm^{-1}) -Raw	0.100 ± 0.001	0.100 ± 0.001	0.100 ± 0.001	0.100 ± 0.001	0.100 ± 0.001	0.100 ± 0.001
UV abs 254nm (cm^{-1}) - Settled	0.085 ± 0.001	0.085 ± 0.001	0.085 ± 0.001	0.085 ± 0.001	0.084 ± 0.001	0.085 ± 0.001
% UV abs removal	16 ± 1	16 ± 1	15 ± 1	16 ± 1	16 ± 1	15 ± 1
DOC (mg/L) - Raw	4.60 ± 0.00	4.60 ± 0.00	4.60 ± 0.00	4.60 ± 0.00	4.60 ± 0.00	4.60 ± 0.00
DOC (mg/L) - Settled	4.37 ± 0.06	4.33 ± 0.15	4.30 ± 0.10	4.27 ± 0.12	4.20 ± 0.00	4.37 ± 0.06
% DOC removal	5 ± 1	6 ± 3	6 ± 2	7 ± 3	9	5 ± 1

***Data shows the mean and standards deviation from three replicate experiments.**
